# Psychometric investigation of the Chinese version of the Habit, Reward and Fear Scale (HRFS)

**DOI:** 10.1002/brb3.2364

**Published:** 2021-09-23

**Authors:** Tu Hong, Chuan He, Zhong‐ke Gu, Jun‐jie Xie, Qian Lu, Yong‐Qiang Li, Xing‐Jun Xu, Ying Shen, Yun‐Qiang Wang, Hui Zheng

**Affiliations:** ^1^ Department of Rehabilitation Medicine The Affiliated Jiangsu Shengze Hospital of Nanjing Medical University Suzhou China; ^2^ College of Psychology Nanjing Normal University Nanjing China; ^3^ Department of Sport and Health Sciences Nanjing Sport Institute Nanjing China; ^4^ Rehabilitation Medicine Center The First Affiliated Hospital of Nanjing Medical University Nanjing China; ^5^ Shanghai Key Laboratory of Psychotic Disorders Shanghai Mental Health Center Shanghai Jiao Tong University School of Medicine Shanghai China

**Keywords:** carving, motivation, nicotine dependence, psychometric testing, questionnaires, tobacco smoking

## Abstract

**Background:**

Tobacco use is one of the most important risk factors for health, and China is the largest producer and consumer of tobacco in the world. Monitoring and controlling the tobacco epidemic is an important issue. However, the motivation underlying smoking behavior is complex and specific to the individual. The Habit, Reward and Fear Scale (HRFS) is a feasible tool to evaluate this complex motivation.

**Objectives:**

To validate the psychometric properties of the HRFS Chinese version (HRFS‐C) and to assess the relationship between motivation and smoking behavior.

**Method:**

We recruited 967 participants through social media and assessed their smoking behavior with three instruments: the Fagerstrom Test for Nicotine Dependence‐Chinese version (FTND‐C), the Questionnaire on Smoking Urges‐Brief Scale‐Chinese version (QSU‐brief‐C), and the HRFS‐C. Ultimately, we retained 700 valid data points. Cronbach's *α* and split‐half tests were used to evaluate the reliability. Confirmatory factor analysis, Pearson's *r* and an analysis of variance (ANOVA) were used to evaluate the validity. In addition, linear regression was used to explore the relationship among the three instruments. The HRFS‐C showed good homogeneity (*α* = 0.965), concurrent validity, and discriminant validity. A significant linear relationship was observed among the FTND‐C, QSU‐brief‐C, and HRFS‐C (*p* < .001).

**Conclusion:**

The motivation measured by the HRFS‐C can significantly predict nicotine dependence and craving in the smoking population. The HRFS‐C can be used to carry out targeted interventions for addicted patients (e.g., motivational enhancement therapy).

## Graphical abstract

HRFS‐C is a good and potential instrument for measuring the motivation of substance use disorder. Motivation measured by HRFS‐C can significantly predict nicotine dependence and tobacco craving.

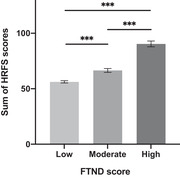

## 1 INTRODUCTION

Tobacco smoking continues to be a major public health problem worldwide. More than 7 million smokers have died from direct tobacco use, and 1.2 million non‐smokers have died due to exposure to second‐hand smoke (World Health Organization, 2019). In the entire 20th century, approximately 1 billion people died prematurely because of tobacco use. China is the largest producer and consumer of tobacco in the world; approximately 50% of Chinese male adults and approximately one million individuals die from tobacco‐related diseases each year (World Health Organization, 2017).

Although most smokers are aware of the harmful effects of smoking and have a strong incentive to quit, only 2−5% of smokers are abstinent for the first year after quitting (Luijten et al., 2020). There is evidence that tobacco abstinence or reduction in smoking will increase the carving for tobacco and lead to a strong motivation to smoke, and this strong motivation to smoke will destroy tobacco abstinence especially in people with high nicotine dependence (Darlow & Lobel, 2012; Yuan et al., 2017). Thus, to improve abstinence among individuals who want to quit smoking, we must understand the motivations for tobacco use.

The behavioral motivations underlying substance use disorders are complex (Sher et al., 2015). There are two important forms of reinforcement related to motivation: positive reinforcement, which is related to expectations of rewarding outcomes (e.g., euphoria), and negative reinforcement, which is related to a fear of negative outcomes (e.g., withdrawal) (Koob & Volkow, 2016). Over the past two decades, habitual behavior and its relationship with motivation have been studied in the field of substance use disorders (Everitt & Robbins, 2005). Goal‐directed and habitual behaviors have often been used to explain the motivations behind substance use disorders (Everitt & Robbins, 2016). Habitual substance seeking is an important motivation for individuals with substance use disorders, and it is usually observed at later stages of the disorder (Yuan, Yu, et al., 2018). In other words, reward, fear, and habit can be used as three dimensions to explain the motivation for substance use.

Reward, fear, and habit have also been used in recent studies to explain the motivation for tobacco smoking. Researchers have suggested that the intention to seek rewarding outcomes can significantly increase tobacco cravings (Liu et al., 2021). In addition, some evidence has indicated that this intention for reward can make quitters start smoking again (Dijkstra & Borland, 2003; Yuan, Zhao, et al., 2018). Similarly, negative reinforcement, especially the alleviation of withdrawal‐related negative emotions, is also a key motivation for smoking (Hall et al., 2015). There is evidence that quitters with social anxiety are more likely to start smoking again (Bakhshaie et al., 2018). Researchers also found that a fear of obesity tends to disrupt the process of quitting smoking in the smoking population (Bush et al., 2014). In addition, there is empirical evidence that negative emotions (such as fear) can increase tobacco cravings, with significant sex differences observed (Perkins et al., 2013). Further, habitual motivation is an important factor; habit‐driven smoking behavior is the primary smoking behavior of heavy smokers (Wiers et al., 2013). According to the complexity and individual differences of smoking motivation (Yuan et al., 2016), a stable instrument that can accommodate and distinguish these three dimensions (reward, fear, and habit) is needed.

A self‐report scale study of alcohol‐seeking motivation in people with alcohol use disorder has been suggested (Piquet‐Pessoa et al., 2019). Based on the above three dimensions, the Habit, Reward and Fear Scale (HRFS) was developed, comprising 18 items to measure the key motivations (habit, reward, fear) behind substance use disorders (Piquet‐Pessoa et al., 2019). The HRFS score of an individual is indicative of the degree of their motivation, and their scores on different subdimensions represent their dependence on different motivation components. However, the psychometric attributes of the HRFS‐Chinese version (HRFS‐C) have not been verified. Thus, in the current study, we aimed to examine the reliability and validity of the HRFS‐C. We hypothesized that the HRFS‐C score can predict the severity of tobacco smoking in a population from East China.

## 2 METHOD

### 2.1 Study design

The study aims to translate the HRFS into Chinese and to evaluate the psychometric properties, including the reliability and validity of the HRFS‐C, and assess self‐reported drivers of tobacco use among adults in southeastern China. In this study, all the participants completed three scales, including the Fagerstrom Test for Nicotine Dependence Scale (FTND‐C), the Chinese version of the Questionnaire on Smoking Urges‐Brief Scale (QSU‐brief‐C), and the HRFS‐C.

### 2.2 Participants

We recruited participants through a mainstream social media (WeChat) in China. We explained the research process to ensure that participants understood the details of the research. All participants volunteered to participate and completed online questionnaires. After the answer is completed, there is a chance that volunteers will be rewarded with a similar time cost (20–40 RMB). Most of the participants were from universities in Nanjing, Jiangsu Province, China. We screened out the participants by self‐reported smoking amount, and the participants who smoked more than zero cigarettes per day were retained. We collected a total of 967 questionnaires during 2019. According to the above screening criteria, the information of 239 non‐smoking participants was screened out, and 28 participants were screened out due to data loss. Finally, we obtained 700 valid questionnaires.

## 3 ASSESSMENTS

The instruments used in this study to measure the tobacco cravings of the participants were the HRFS, the FTND, and the QSU‐brief.

### 3.1 Habit, Reward, and Fear Scale

The HRFS, developed by Marcelo Piquet‐Pessoa et al. (2019), consists of 18 items to assess motivations for obsessive‐compulsive and substance use disorders. This scale has three original dimensions: 6 habit‐related items are used to qualify non‐affective motivation, and 6 reward‐related and 6 fear‐related items are used to qualify affective motivation. The score for answers to each item ranged from 1 (disagree) to 7 (agree). This scale was first used in patients with alcohol use disorder, and it achieved satisfactory psychometric characteristics. Two graduate students translated the HRFS, and a doctoral student in clinical psychiatry integrated their work. Finally, the HRFS‐C was tested in patients and revised by a physician in the substance dependence department.

### 3.2 Fagerstrom Test for Nicotine Dependence

We used the FTND to evaluate the participants’ nicotine‐dependence‐related symptoms. Items 1 and 4 were scored from 0 to 3, and the remaining four items were scored as 0 or 1. A higher score on the FTND indicates greater nicotine dependence (Heatherton et al., 1991). The FTND is widely used in clinical studies to assess nicotine dependence, and Cronbach's *α* of FTND‐C was 0.74 in a previous study (Huang et al., 2006). In this study, Cronbach's *α* was 0.604.

### 3.3 Questionnaire on Smoking Urges‐Brief Scale (QSU‐brief)

The QSU‐brief contains 10 items scored on a 7‐point Likert‐type scale. Subjects evaluate the statements on a scale from 1 (strongly disagree) to 7 (strongly agree) based on their current feelings to assess their craving for tobacco (Toll et al., 2004). The reliability of the QSU‐brief‐C has been verified in previous studies (Yu et al., 2010).

### 3.4 Demographic characteristics

In terms of demographic variables, age, sex, years of education, and daily smoking amount of the participants were collected, and a daily smoking amount was used as the main indicator for screening the participants.

## 4 DATA ANALYSIS

Statistical analysis was performed by Jamovi version 1.1 (www.jamovi.org). We used descriptive statistical indicators, including frequency distribution, mean, and standard deviation, to describe the demographic variables of the subject population. Homogeneity (Cronbach's *α*) was used to examine the reliability. We used confirmatory factor analysis (CFA) to verify the three dimensions of the HRFS envisaged by the original author, namely, habit, reward and fear. We also examined the correlation between the HRFS score and the FTND and the QSU‐brief scores to explain the correlation validity by Pearson's correlation. Finally, the participants were divided into three groups according to their scores on the FTND, and the discriminant validity of the HRFS was investigated by an analysis of variance (ANOVA) with the least significant difference (LSD) for multiple‐comparison tests.

Linear regression was used to explore the relationship between motivation (measured by the HRFS) and nicotine dependence (measured by the FTND) and craving (measured by the QSU‐brief). In linear regression, scores on the FTND and QSU‐brief were taken as dependent variables, while sex, age, and HRFS score were taken as the main independent variables. After we performed linear regression, the trend test (*p* for trend) was used to verify the linear relationship between variables.

## 5 RESULTS

### 5.1 Demographic data

We retained 700 participants who self‐reported smoking more than zero cigarettes per day. Among all participants, the average age was 29.90 (SD 10.70) years, ranging from 18 to 60 years; 80.7% (*n* = 565) were male, and 19.7% (*n* = 135) were female. These participants smoked 8.82 (8.19) cigarettes per day on average, and we found a significant difference between the sexes (*p *< .001). The FTND score of these participants was 2.23 (2.22). Most of them had some college education, with an average of 13.40 (2.47) years of education (Table 1).

**TABLE 1:** Demographic variables

		Sample size	Weight	Age Mean (SD)	Education years Mean (SD)	Cigarettes‐days Mean (SD)
Total sample		700	100%	29.92 (10.70)	13.40 (2.47)	8.82 (8.19)
Sex						
	Male	565	81%	29.00 (10.20)	13.50 (2.37)	9.64 (8.53)
	Female	135	19%	33.9 (11.90)	13.20 (2.86)	5.41 (5.42)

*Abbreviation*: SD = standard deviation.

**TABLE 2:** Fit indexes for the confirmatory factor models of the HRFS

*χ* ^2^	df	CFI	TLI	SRMR	RMSEA (RMSEA 90% CI)
2016***	132	0.84	0.815	0.0637	0.143 (0.137–0.148)

*Note*: *N* = 700 for the chi‐square analysis.

*Abbreviation*: CFI, comparative fit index; HRFS, Habit, Reward and Fear Scale; RMSEA, root‐mean‐square error of approximation; SRMR, standardized root‐mean‐square residual; TLI, Tucker–Lewis index.

*** *p *< .001.

### 5.2 Reliability

Cronbach's *α* coefficient for the overall HRFS was 0.965, and in the subdimensions, Cronbach's *α* coefficients were 0.922 (habit), 0.910 (reward), and 0.880 (fear). The Spearman–Brown length‐related coefficient was 0.946, and the Guttman split‐half coefficient was 0.946. According to the criterion for clinical scale, the HRFS‐C has good reliability (Henson, 2019).

### 5.3 Construct validity

CFA (*n* = 700) was used to verify the construct validity of the HRFS‐C based on the three‐factor structure of the original English version. Items 3, 6, 7, 10, 14, and 16 belong to the dimension of habit; items 2, 4, 9, 12, 15, and 17 belong to the dimension of reward; and items 1, 5, 8, 11, 13, and 18 belong to the dimension of fear. Chi‐square values and the comparative fit index (CFI), Tucker–Lewis index (TLI), standardized root‐mean‐square residual (SRMR), and root‐mean‐square error of approximation (RMSEA) were used to assess the model fitness. According to the criterion of model fitting indexes, the CFI and TLI should be close to 0.95, the SRMR should be close to 0.08 and the RMSEA should be close to 0.06 (Hu & Bentler, 1999). The HRFS‐C did not fit the three‐factor model of the English version well (Table 2).

### 5.4 Concurrent validity

We evaluated the concurrent validity of the HRFS by calculating Pearson's coefficient with the FTND and the QSU‐brief. Pearson's coefficient was .465, *p *< .01 (between the HRFS and the FTND), and .525, *p *< .01 (between the HRFS and the QSU‐brief, Table 3).

**TABLE 3:** Correlation matrix

		FTND	QSU	HRFS
FTND	Pearson's *r*	—		
QSU‐brief	Pearson's *r*	0.436***	—	
HRFS	Pearson's *r*	0.465***	0.525***	—

*** *p *< .001.

### 5.5 Discriminant validity

**FIGURE 1:**
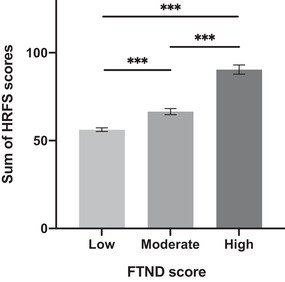
Significant differences in HRFS scores among the three groups divided by FTND scores. Low = 0 to 2 points on the FTND; Moderate = 3 to 6 points on the FTND; High = 6 to 10 points on the FTND; *Abbreviations*: FTND, Fagerstrom Test for Nicotine Dependence Scale; HRFS, Habit, Reward and Fear Scale. ***p < .001

One‐way ANOVA was used to determine whether there were significant differences between the groups with different levels of tobacco use disorder. According to the original FTND scoring manual, five levels of nicotine dependence were identified: very low (0 to 2 points), low (3 to 4 points), moderate (5 points), high (6 to 7 points), and very high (8 to 10 points). However, empirical evidence suggested that FTND scores for regular smokers were greater than 3 (Huang et al., 2008), while scores for heavy smokers were greater than 6 (de Leon et al., 2003). According to these recommended cut‐off scores, we divided the participants into three groups—those with low dependence (0 to 3 points; *n* = 430), moderate dependence (3 to 6 points; *n* = 203), and high dependence (6 to 10 points; *n* = 67). We found a significant difference among the three groups (*F* = 78.1, *p *< .01, *η* ^2^ = .02; Figure 1).

## 6 REGRESSION RESULTS

QSU‐brief scores were used as the predicted variable; HRFS score, sex, and age were used as predictor variables in the equation by the stepwise method; and two linear models of QSU‐brief score were used as Y variables. Since the coefficient is not significant (*p *> .05), sex was removed from the equation. According to the results, the second model is more suitable (Table 4).

**TABLE 4:** Regression Coefficients of QSU‐brief

		*B*	SE *B*	*β*	*R* ^2^	Δ*R* ^2^	*F*
Model 1					0.275	0.275	265.199***
	(Constant)	1.732	2.1				
	HRFS	0.509	0.031	0.525***			
Model 2					0.289	0.014	141.556***
	(Constant)	5.559	2.709				
	HRFS	0.49	0.031	0.505***			
	Age	0.263	0.072	0.118***			

*Note*: Dependent variable: QSU‐brief.

*** *p *< .001.

Similarly, we took the FTND score as the predicted variable and input HRFS score, sex, and age as the predictor variables into the equation by a stepwise method. Sex (*p *> .05) was removed from the equation. Of the two linear models we established, the second model was more suitable (Table 5).

**TABLE 5:** Regression Coefficients of FTND

		*B*	SE *B*	*β*	*R* ^2^	Δ*R* ^2^	*F*
Model 1					0.216	0.216	192.138***
	(Constant)	−0.375	0.202				
	HRFS	0.042	0.003	0.465***			
Model 2					0.244	0.029	112.729***
	(Constant)	−1.272	0.264				
	HRFS	0.039	0.003	0.436***			
	Age	0.035	0.007	0.171***			

*Note*: Dependent variable: FTND.

*** *p *< .001.

### 6.1 Trend test results

After we performed linear regression, we used trend tests to verify the linear relationship among the variables above. HRFS score quartiles were used to divide participants into four groups, with the median of each group used for trend testing. According to the results in Table 6, the linear relationship among the above variables was significant.

**TABLE 6:** *β* of HRFS score quartiles in regression with QSU‐breif and FTND

	Q1 (*n* = 178)	Q2 (*n* = 173)	Q3 (*n* = 186)	Q4 (*n* = 163)	
	(0–46)	(47–65)	(66–78)	(>78)	*p* for trend
**QSU‐brief**					
Model 1	Reference	.111	.335	.570	<.001
*p*‐Value		.005	<.001	<.001	
Model 2	Reference	.112	.329	.565	<.001
*p*‐Value		.005	<.001	<.001	
Model 3	Reference	.100	.319	.544	<.001
*p*‐Value		.011	<.001	<.001	
**FTND**					
Model 1	Reference	.090	.207	.513	<.001
*p*‐Value		.029	<.001	<.001	
Model 2	Reference	.091	.201	.508	<.001
*p*‐Value		.028	<.001	<.001	
Model 3	Reference	.074	.188	.477	<.001
*p*‐Value		.069	<.001	<.001	

*Note*: Model 1 was adjusted for HRFS score; Model 2 was adjusted for HRFS score and sex; Model 3 was adjusted for HRFS score, sex, and age. The test for trend was based on variables containing the median value for each quartile.

## 7 DISCUSSION

This study of the psychometric properties of the 18‐item HRFS in smoking adults from East China revealed that the HRFS‐C was satisfactory to assess the motivation of smoking behavior among Chinese individuals. First, the satisfactory homogeneity together with the split‐half coefficient indicated that the HRFS‐C is reliable, which is consistent with the results of previous studies on the original English HRFS (Piquet‐Pessoa et al., 2019). Second, the results of CFA showed that the HRFS‐C did not fit the three dimensions envisaged in the original version well. Third, an analysis of variance revealed that the HRFS‐C score was able to distinguish participants with different levels of nicotine dependence, indicating the good discriminant validity of the HRFS‐C. Finally, the correlation coefficient and regression analysis suggested that the HRFS‐C score could be used as a predictor of cravings and nicotine dependence in the smoking population. These statistical results confirmed our hypothesis that the HRFS‐C score can be used to predict the severity of tobacco smoking in China.

The HRFS‐C has certain psychometric properties that are related to those of its original construct. In the original HRFS, 18 items were grouped into three dimensions: habit, reward, and fear. These three factors have been confirmed to explain the motivation of people with substance use disorders (Buckner et al., 2011; Morean et al., 2018; Volkow & Morales, 2015). Moreover, the items under each dimension were selected and modified from scales with verified psychometric properties (Verplanken & Orbell, 2003). According to the good fit indices, CFI and TLI can be considered as marginal fit, SRMR can be considered as acceptable fit, and RMSEA can be considered as bad fit (Hu & Bentler, 1999). In general, the results of CFA were not satisfactory. In terms of this sample, we found that the factor loadings of item 1 (0.57 < 0.7) and item 2 (0.68 < 0.7) are at a low level, and the *R* ^2^ of item 1 (.32 < .4) was not good enough (MacCallum et al., 2001). We believe that the existence of these two items has an impact on the reliability of the structure of HRFS, and attention should be paid to these items in other samples or fields during the revision process. We suspect that the difference in expression between the two languages might lead to a decrease in the differentiation of the three dimensions of the HRFS‐C.

Another interesting finding was that the HRFS‐C score could significantly predict cravings and the severity of nicotine dependence. In the regression model, we found a reliable linear relationship between nicotine dependence, carving, and motivation measured by HRFS. The relationships between motivation, craving, and nicotine dependence are consistent with a previous study (Reese & Veilleux, 2016). This evidence significantly indicated that despite the unsatisfactory structure of HRFS in this sample, the total score of HRFS can still reflect the intensity of motivation and subsequently predict the severity of tobacco dependence. Moreover, similar results have been found in studies of alcohol use disorders. Higher motivation was associated with higher alcohol cravings and alcohol dependence (Blaine et al., 2019). Besides, there is evidence that motivation can be a predictor of alcohol cravings (Pombo et al., 2016). The results of this study and the use of the HRFS in people with obsessive‐compulsive disorder and alcohol use disorders (Ferreira et al., 2020) suggest the potential clinical value of the HRFS‐C. In general, HRFS provides a theoretical motivation model that can be used to understand different forms of psychopathology, such as substance use disorders, behavioral disorders, and obsessive‐compulsive related disorders.

However, there are some limitations in this study that can be improved in future studies. First, the age and sex of the participants were not well matched. Although the age of participants covered all adult age groups, the majority of participants were approximately 20 years old, which resulted in a large standard deviation and affected our data distribution. Due to the limited data sources, we failed to match the sex of the smoking population, which affected the external validity of our results. Second, the structure of the HRFS‐C was not clarified in this study. The results of CFA indicated that the three dimensions envisaged by the original version were not the best model of the HRFS‐C. Thus, the HRFS‐C failed to distinguish between different types of motivations. Third, we lacked the back‐translation procedure when translating the HRFS, and the findings in this paper need to be considered with caution. In order to remedy this defect, we re‐translated the HRFS by combined translation technique (Cha et al., 2007) and compared it with the previous version. We believe that the Chinese version of HRFS is valid in the present study.

In conclusion, the HRFS‐C has good reliability, discriminant validity, and concurrent validity. In the future, this scale could be used to reveal the motivation domains of other substance use disorders.

## AUTHOR CONTRIBUTIONS

Ying Shen, Yun‐Qiang Wang, and Hui Zheng designed the study; Qian Lu, Zhng‐ke Gu, Yong‐Qiang Li, and Xing‐Jun Xu performed the study; Tu Hong and Jun‐jie Xie analyzed the results; Tu Hong and Chuan He wrote the paper together; all authors have read and approved the final version of the manuscript.

### TRANSPARENT PEER REVIEW

The peer review history for this article is available at https://publons.com/publon/10.1002/brb3.2364

## Data Availability

The datasets for this study can be found in the OSF: osf.io/4zdm5
